# Formulation and Evaluation of Amikacin Sulfate Loaded Dextran Nanoparticles against Human Pathogenic Bacteria

**DOI:** 10.3390/pharmaceutics15041082

**Published:** 2023-03-28

**Authors:** Rahamat Unissa Syed, Sivakumar S. Moni, Muhammad Nawaz, Mohammed Khaled Bin Break, Nasrin E. Khalifa, Siddig Ibrahim Abdelwahab, Reham Meshal Alharbi, Raghad Huraid Alfaisal, Bayan Naif Al Basher, Entsar Mohammed Alhaidan

**Affiliations:** 1 Department of Pharmaceutics, College of Pharmacy, University of Ha’il, Hail 81442, Saudi Arabia; n.aldirdiri@uoh.edu.sa; 2 Medical and Diagnostic Research Centre, University of Ha’il, Hail 55473, Saudi Arabia; m.binbreak@uoh.edu.sa; 3 Department of Pharmaceutics, College of Pharmacy, Jazan University, Jazan 45142, Saudi Arabia; 4 Department of Nano-Medicine Research, Institute for Research and Medical Consultations, Imam Abdulrahman Bin Faisal University, Dammam 31441, Saudi Arabia; nawwaz@gmail.com; 5 Department of Pharmaceutical Chemistry, College of Pharmacy, University of Ha’il, Hail 81442, Saudi Arabia; 6 Department of Pharmaceutics, Faculty of Pharmacy, University of Khartoum, Khartoum 11115, Sudan; 7 Medical Research Center, Jazan University, Jazan 45142, Saudi Arabia; siddigroa@yahoo.com; 8 College of Pharmacy, University of Ha’il, Hail 81442, Saudi Arabia; rehameshalh@gmail.com (R.M.A.); raghad.01299@gmail.com (R.H.A.); naefbayan66@gmail.com (B.N.A.B.); entsarm72@gmail.com (E.M.A.)

**Keywords:** dextran sulfate sodium, Amikacin, Nanoparticles, physical characterization, antibacterial effect, human pathogenic bacteria

## Abstract

Amikacin sulfate-loaded dextran sulfate sodium nanoparticles were formulated, lyophilized (LADNP), and then analyzed. The LADNP had a −20.9 ± 8.35 mV zeta potential, PDI of 0.256, and % PDI of 67.7. The zeta average nano size of LADNP was 317.9 z. d.nm, while the dimension of an individual particle was 259.3 ± 73.52 nm, and nanoparticle conductivity in colloidal solution was 2.36 mS/cm. LADNP has distinct endothermic peaks at temperatures at 165.77 °C, according to differential scanning calorimetry (DSC). The thermogravimetric analysis (TGA) showed the weight loss of LADNP, which was observed as 95% at 210.78 °C. XRD investigation on LADNP exhibited distinct peaks at 2*θ* as 9.6°, 10.4°, 11.4°, 18.9°, 20.3°, 24.4°, 28.2°, 33.2°, 38.9°, and 40.4° confirming crystalline structure. The amikacin release kinetics from LADNP revealed zero order kinetics with a linear release showed zero order kinetics with 37% of drug release in 7 h and had an R^2^ value of 0.99. The antibacterial effect of LADNP showed broad-spectrum activity against tested human pathogenic bacteria. The preset study demonstrated that LADNP is a promising antibacterial agent.

## 1. Introduction

Amikacin sulfate is an aminoglycoside antibiotic that is typically used widely for the treatment of Gram-negative infections [1]. According to World Health Organization recommendations, the aminoglycoside antibiotic amikacin is frequently recommended to patients with multiple drug-resistant tuberculosis and Gram-negative bacterial infections. However, amikacin can cause severe nephrotoxicity, neurotoxicity, and ototoxicity [2]. In contrast, kidney diseases are becoming an increasingly serious public health issue all over the world, including in Saudi Arabia. The number of people in the Kingdom suffering from chronic renal problems has increased by 25.1% over the course of the previous decade. According to the findings of the research, the prevalence of chronic kidney disease (CKD) in Hail is 75% [3,4]. Furthermore, bacterial resistance to antibiotics is a significant threat and challenge for researchers to overcome therapeutic incompatibility caused by the expression of resistant factors. The irrational utilization of antibiotics is a primary drive for the emergence of resistance across the world, especially in developing countries, because of a lack of proper knowledge and understanding of the appropriate usage of antibiotics [5,6]. On the other hand, amikacin is rapidly metabolized and eliminated as such via glomerular filtration, resulting in a plasma t½ of around 2 h in individuals with normal renal function. In individuals who have renal failure, the half-life of amikacin may be extended to anywhere between 30 and 60 h [7]. As a result, individuals who have renal failure require a dosage reduction, which is also more significant from a therapeutic standpoint. The most innovative technology, nanotechnology, can offer the correct tools for drug effectiveness, targeted distribution to cells, bypass harmful effects, and boosting therapeutic compatibility. In the current therapeutic era, delivering antibiotics at the cellular level of bacteria and overcoming these resistant pathways has become a formidable problem for researchers. These factors have prompted pharmaceutical scientists to engage in the development of new drug delivery systems, especially to overcome multiple drug resistance. Therefore, the purpose of this study is to design and formulate a nanoparticle system for amikacin delivery using dextran sulfate sodium. 

## 2. Materials and Methods

### 2.1. Materials

Dextran sulfate (sodium salt, MW = 500,000) and sodium tripolyphosphate (MW = 367.86) were purchased from Santa Cruz Biotechnology, Inc., Dallas, TX, USA. Amikacin sulfate was purchased from Sigma-Aldrich, St. Louis, MO, USA. Bacteriological media and other chemicals and solvents were purchased from Scharlau, Spain. Ejadah Medical Supplies Est, Riyadh, Saudi Arabia, supplied all the materials used in this research. 

### 2.2. Formulation of Nanoparticles

Amikacin sulfate-loaded dextran nanoparticles (ADNP) was prepared using an ionic gelation technique with a modified approach [7,8]. Briefly, dextran sodium sulfate solution (2% *w/v*) was prepared in Milli Q water. The solution was kept stirred on a hot plate with a magnetic bead. The speed was regulated at 2000 rpm for about 3 h. The 1 mL of 1% (*w/v*) tripolyphosphate was prepared in Milli Q water and added as a chemical cross-linker initially after running for about 30 min. That reacting solution is called a reaction mixture (RM). Then after 1% (*w/v*), tripolyphosphate was added during the formulation process at a predetermined time interval in RM. At periodic intervals, 1 mL of 1% (*w/v*) amikacin sulfate was added dropwise to the RM and ran for 15 min. During the formulation of nanoparticles, sonication was conducted for 3 min twice at predetermined time intervals. Finally, the RM was filtered through a 0.2 µm PVDF membrane, and the filtrate was subjected to various analyses. 

### 2.3. Lyophilization Process

The lyophilization was performed by using a Millrock BT85 desktop freeze dryer (Millrock Technology, Kingston, NY, USA). A reaction mixture was created in a glass flask by mixing ADNP with 6% *w/v* mannitol in a 1:1 volume ratio. For freeze-drying, the mixture was kept in a deep freezer at −80 °C for 24 h. After that, the ADNP was put in vacuum-controlled lyophilizing tubes. The temperature was kept at −84 °C, and the vacuum pressure was kept at 3000 pascals. The lyophilized ADNP (LADNP) was eluted from the glass flask after 24 h, then pooled and stored at 4 °C for further analysis.

### 2.4. Dynamic Light Scattering (DLS) Analysis

The nanoparticles were physically characterized by measuring their zeta potential (ZP) in millivolts (mV), their conductivity in milli siemens per centimeter (mS/cm), their size in nanometers (d.nm and z.dnm), and their polydispersity index (PDI). Briefly, 5% *w/v* of LADNP was prepared in Milli Q water, and placed in capillary cells, and physical characterization of nanoparticles was performed by using Zetasizer Nano NS, Malvern Instruments, Malvern, UK [6].

### 2.5. Determination of Morphological Features

The morphological features and particle size of the formulated nanoparticles were studied using a high-resolution scanning electron microscopy study. Using a VEGA3 TESCAN (Czech Republic) scanning electron microscopy (SEM) with high resolution, the morphological properties of LADNP were analyzed. The morphological properties of LADNP were analyzed by Transmission electron microscopy (TEM), FEI, Morgagni 268, Brno, Czech Republic. 

### 2.6. Energy Dispersive Spectroscopy (EDAX) Analysis

Energy Dispersive Spectroscopy (EDAX) is a useful technique for determining the elemental compositions of samples. The EDAX spectrum of LADNP was obtained by using EDAX APEX, AMETEK, USA. The acceleration voltage was set at 5 keV, and the data were recorded after 31 s.

### 2.7. Differential Scanning Calorimetry (DSC) Analysis

LADNP were analyzed by means of the DSC method to ascertain the enthalpy changes that occurred because of changes in the physical and chemical properties of the samples. DSC analysis was performed by Shimadzu DSC 60 (Nakagyo-ku, Kyoto, Japan)). In a non-hermetically sealed aluminum pan, LADNP was placed, and the temperature was increased from 30 to 250 °C at a rate of 10 °C per minute while maintaining a 10 mL/min airflow min^−1^.

### 2.8. Thermogravimetric ANALYSIS (TGA)

The thermal stability of the samples in an air atmosphere was assessed using a thermogravimetric analyzer (Shimadzu thermo gravimetric analyzer) was utilized to determine the thermal stability of LADNP. After placing the LADNP (10 mg) on an aluminum pan, the test was carried out at a heating rate of 10 °C per minute within a temperature range of 50 °C to 300 °C. 

### 2.9. X-ray Diffraction (XRD) Analysis

X-ray diffraction (XRD) was utilized so that the crystalline structures of LADNP could be investigated. The LADNP was subjected to XRD examination using X-ray diffraction (Rigaku, Japan. The XRD diffractograms at 2*θ* in the range of 2–80° at a voltage of 45 kV and a current of 0.8 mA were produced using Cu K α radiation from the incoming beam (λ = 1.5418 Å). A scanning range of 2*θ/θ* was selected, and a scanning speed of 10 min^−1^ was employed.

### 2.10. Loading and In Vitro Release Study

#### 2.10.1. Preparation and Validation of Standard Cure

Amikacin sulfate powder was dissolved in 10 mL of Millipore water to create a working stock solution (1000 g/mL). Then, using a serial dilution of the stock standard solution, working standard solutions with concentrations of 500, 250, 125, 62.5, 31, 25, 15.6, and 7.8 g/mL was created in Millipore water. The calibration curve was created by measuring the absorbance of the produced standard dilutions at four different wavelengths and comparing them to a transparent blank (250, 265, 290, 310, 380, and 405 nm) in a UV/visible spectrophotometer. In order to validate the method, linearity was determined at several wavelengths. Linearity at these wavelengths indicates that the method is consistent with Beer-law. Lambert’s Plotting the absorbance values at max against the amounts of amikacin sulfate allowed for the construction of the standard curve. 

#### 2.10.2. Loading Study 

The release of amikacin sulfate from LADNP was determined by establishing a standard curve. Entrapped amikacin sulfate was released after being suspended for 30 min in 10 mL of 0.1 N HCl solution containing 5 g of LADNP. After centrifuging the reaction mixture at 3000 RPM, the supernatant was collected and kept at a temperature of 2 °C. The amikacin sulfate concentration was estimated from the standard curve. Then, using the following equations, the drug loading (DL) was calculated:

$$\mathrm{DL}\;\left(\%\right)=\frac{\mathrm{Total}\;\mathrm{amount}\;\mathrm{of}\;\mathrm{amikacin}\;\mathrm{sulfate}\;\mathrm{extracted}\;\mathrm{from}\;\mathrm{the}\;\mathrm{nanoparticles}}{\mathrm{Total}\;\mathrm{weight}\;\mathrm{of}\;\mathrm{amikacin}\;\mathrm{sulfate}\text{-}\mathrm{loaded}\;\mathrm{nanoparticles}}\times 100$$

#### 2.10.3. In Vitro Release Profile

100 mg of LADNP was placed in a dialysis bag and immersed in 50 mL of phosphate buffer saline (pH 7.4/37 °C) with a magnetic bead swirling at 1000 rpm for 7 h. The burst release phase was assessed after 30 min. After that, 3 mL of medium was removed from the tube every hour. The samples were analyzed using UV/visible spectroscopy in Shimadzu, Japan. The release pattern was determined by extrapolating the optical density against amikacin concentration.

### 2.11. In Vitro Antibacterial Study

#### 2.11.1. Bacterial Strains Used and Standardization of Bacterial Cultures

The bacterial strains used in the study were Staphylococcus aureus, Staphylococcus epidermidis, Streptococcus pyogenes, Bacillus subtilis, Enterococcus facalis, Escherichia coli, Klebsiella pneumonia, Salmonella cholerasuis, Pseudomonas aruginosa, and Proteus mirabilis. Briefly, 24 h culture was prepared and standardized by gradient dilution from 10^−^^1^ to 10^−^^7^ with nutrient broth. The viability of bacterial culture was identified by assessing colony forming unit per mL (CFU/mL) [8].

#### 2.11.2. Determination of Minimum Inhibitory Concentration

The minimum inhibitory concentration (MIC) of the LADNP for the bacterial species that were examined was calculated using the broth dilution method in accordance with the standard methodology developed by the Clinical and Laboratory Standards Institute, USA [9].

#### 2.11.3. Determination of Antibacterial Susceptibility

Briefly, Muller Hinton agar plates were prepared for conducting the antibacterial study [10]. Bacterial subcultures were prepared from the stock culture, and after 24 h of incubation, the culture was subjected to antibacterial studies. Agar well diffusion technique was performed for both the sample analytes and standard ciprofloxacin disc (50 µg/mL). The inoculation was performed by dipping a sterile cotton swab into the standardized (CFU/mL) culture individually with various organisms and streaked on the MH agar plate by rotating the petri dish to distribute the culture evenly. The plates were allowed to dry for about 10 min before the administration of the sample analyte. The agar well diffusion technique was performed by punching holes on the inoculated MH agar plates using a standard sterile stainless-steel borer. A known concentration (based on the MIC test) of LADNP was placed in the respective wells. The plates were incubated at 37 °C for 24 h, and the development of inhibitory zones assessed the antibacterial spectrum after 24 h of incubation. The spectrum of activity is directly proportional to the diameter of the inhibition zones and tabulated.

### 2.12. Statistical Analysis

Each experiment was conducted three times (n = 3), and a one-way analysis of variance was conducted on the data (ANOVA). The statistical significance levels were at *p* < 0.05 (Significant). Statistical analyses were performed using Prism 9 Graph Pad Instat software system, Boston, MA, USA. Values for the test samples were compared with values for the standard drug using Dunnet’s post hoc test.

## 3. Results and Discussion

### 3.1. Physical and Morphological Characterization

**Figure 1:**
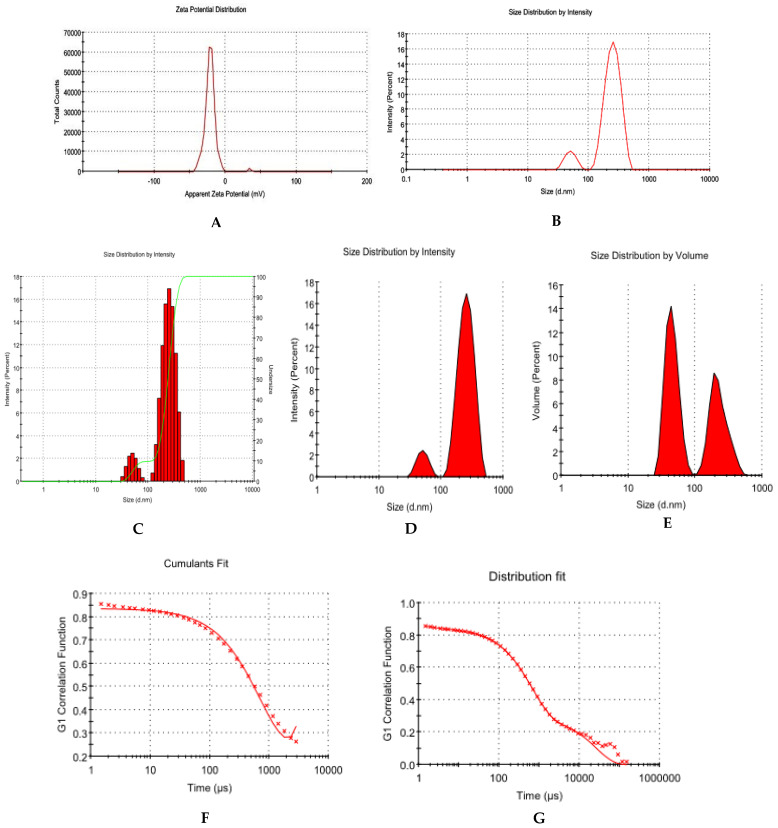
Physical characterization of lyophilized amikacin sulfate loaded dextran nanoparticles. (**A**) Zetapotential analysis (**B**) Size distribution of nanoparticles through particle intensity. (**C**) Various particle size distribution analysis through the percent intensity (**D**) Particle Size distribution analysis through intensity (**E**) Particle Size distribution analysis through volume (**F**) Cumulative fit of particulate colloidal system (**G**) Particle distribution fit of the particulate colloidal system.

The LADNP was in the form of a free-flowing powder, and its physical properties are shown in Table 1. The LADNP showed a good ZP, which was −20.9 ± 8.35 mV with a unique peak. The conductivity was 2.36 mS/cm; thus, the particles had good electrostatic conduction and were electrostatically active (Figure 1A). The LADNP was uniformly formed and highly homogenous in a single phase in the colloidal system, with a PDI value of 0.256 (Figure 1B). The LADNP exhibited 67.7% PDI with 90.2% intensity in a colloidal injectable form. The average zeta particle was observed as 317.9 ± 73.52 nm. The diameter of particles in radius was observed as 259.3 ± 73.52 d.nm with a PD diameter of 215.2 d.nm. According to a previous study, dextran nanoparticles had a zeta average of 69.3 z.d.nm and particle size in terms of radius ranging from 52 to 82 nm [6].

An earlier study found that solid lipid nanoparticles of amikacin had a size of 190.7 d.nm and a ZP of +16 mV. This suggested that the nanoparticles were stable [11]. Recently, amikacin-loaded niosomes had particle sizes that varied from 175.2 to 248.3 nm with a PDI ranging from 0.142 to 0.379 [12]. The surface charge and the size of nanoparticles are two parameters that determine biodistribution and the pharmacokinetic profiles of nanoparticles [13]. The ZP value of −20.9 8.35 mV indicates that the targeting of bacterial cells was conducted in a passive manner. In a colloidal injectable system, nanoparticle mobility is another crucial aspect that determines particle conductivity. Particles with sizes smaller than 20 d.nm have strong mobilities in the colloidal system and can affect the surface charge of particles [6]. According to the findings of this research, particles with a size greater than 200 d.nm exhibit moderate mobility.

The size of the nanoparticle has a role in determining the enhanced permeability and retention (EPR) effect, which is a key component in the process of targeting bacterial cells. Bacteria have varied pore diameters [14]. According to the findings of a previous study, the pores of *S. aureus* ranged in size from 50 to 500 Å, and their diameters fell somewhere between 5 and 50 nm [15]. The cumulative fit analysis revealed a linearity of greater than 99.9%, while the size distribution fit was depicted at approximately 95% in a colloidal dispersion system (Figure 1F,G). The Y-intercept value of the intensity peak is an important tool to estimate the signal:noise ratio of an instrument that measures the particle size intensity of samples and can be used to evaluate data quality. Generally, an ideal signal of a value greater than 0.9 is the best colloidal system [13]. The results of this analysis showed that the 1% *w/v* LADNP preparation had Y-intercept values of 0.854, indicating that it had a good colloidal system.

**Figure 2:**
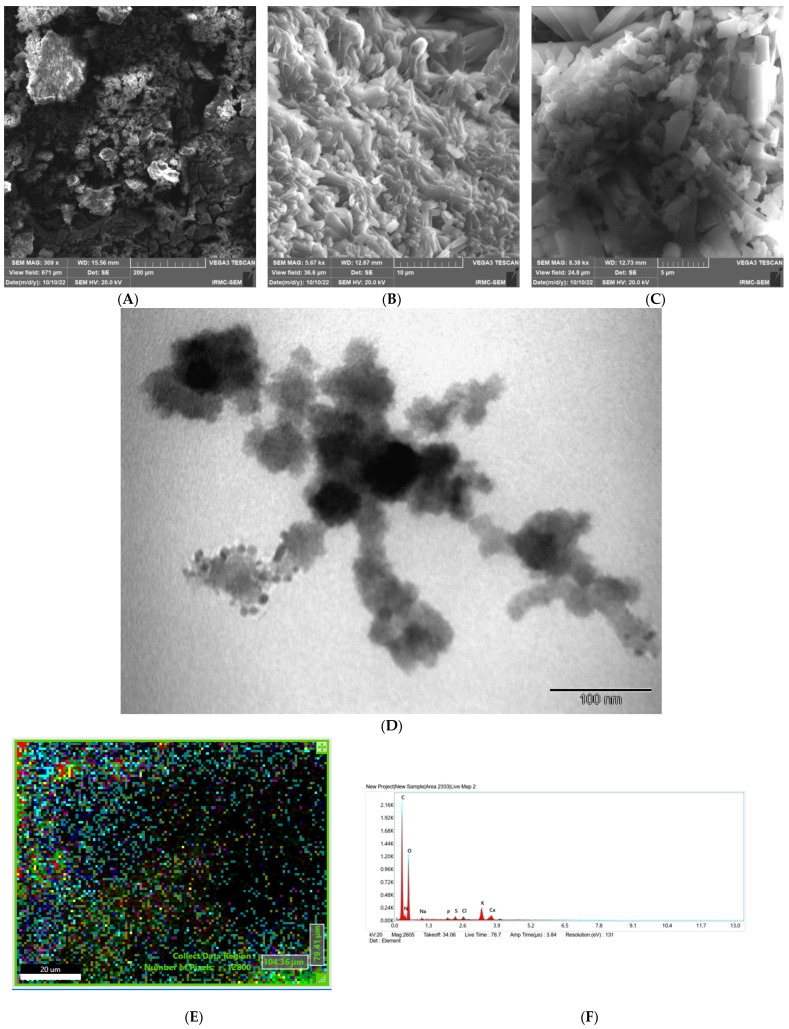
Morphological analysis of lyophilized amikacin sulfate loaded dextran nanoparticles (LADNP)/(**A**) Scanning electron micrograph of LADNP (**B**,**C**) Crystalline structure of LADNP captured using scanning electron microscope (**D**) Transmission electron micrograph of LADNP (**E**) EDAX analysis of LADNP, the area under scanning (**F**) EDAX graph of LADNP.

Figure 2A displays the SEM study on LADNP and indicates particles that are less spherical, and the fact that most of the particles are clumped together may be the result of the lyophilization process. It is essential to emphasize that the lyophilization of nanoparticles may lead to the aggregation of the particles, which has been reported earlier [6]. Crystalline characteristics of particles were observed in some of the particles, as depicted in Figure 2B,C. The TEM results of LADNP are shown in Figure 2D. The LADNP were spherical in shape, with rough surfaces, and the particles were discrete. However, crowded particles were observed with spherical and elongated shapes, as well as ruptured particles, and the particles were of non-uniform sizes. Similarly, dextran particles were also observed to have an irregular shape with spherical and elongated [6]. The energy dispersive X-ray spectrometry (EDAX) analysis of LADNP showed in Figure 2E,F. The study showed that the particle was composed of 51.32% carbon, 36.02% oxygen, and 10.68% of nitrogen, as well as the presence of other trace elements such as Na, K, S, Cl, and Ca.

### 3.2. Thermal Analysis

**Figure 3:**
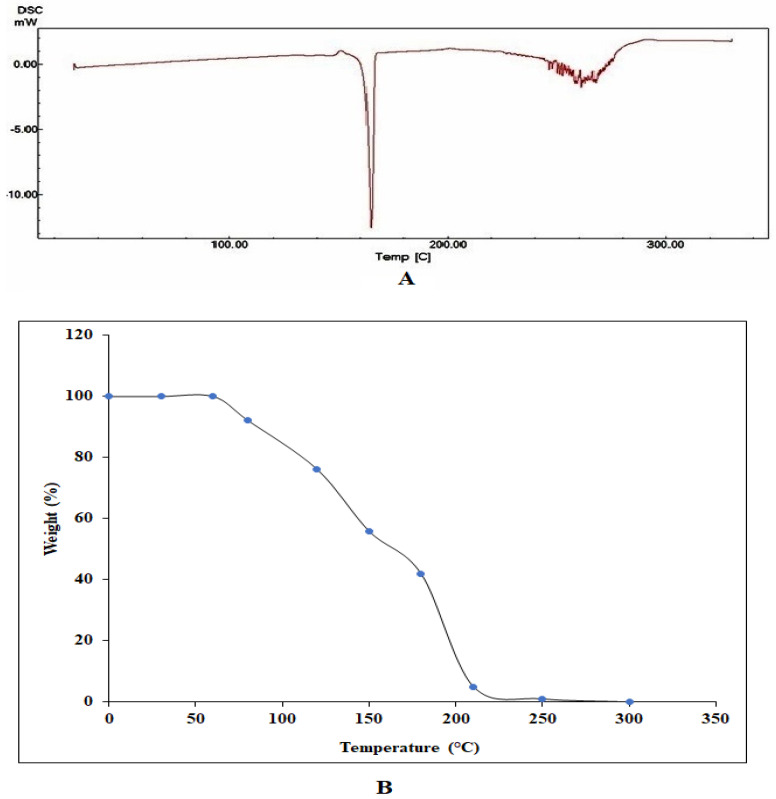
Thermal analysis of lyophilized amikacin sulfate loaded dextran nanoparticles (LADNP). (**A**) Differential scanning calorimetry analysis of LADNP (**B**) Thermogravimetric analysis of LADNP.

Changes in nanoparticle thermodynamic parameters, such as enthalpy, entropy, and heat capacity, produced by physical causes, chemical processes, and phase transitions can be detected using the technique known as differential scanning calorimetry (DSC). The thermal degradation property of LADNP was studied using DSC analysis over a temperature range of 40–360 °C. The glass transition (Tg) temperature was observed from 157.66 to 167.77 °C (Figure 3A). Therefore, LADNP was stable up to 157.66 °C. Previous research reports had revealed that dextran nanoparticles were stable up to 102 °C, and the molecular weight change was observed from 102.82 to 165.77 °C [6]. Similarly, to this, a previous study revealed that the Tg temperature of an iron-oxide dextran-coated magnetic nanoparticle and a pure dextran molecule was approximately 102 °C. The same study also showed that when dextran was adsorbed on magnetic nanoparticles, dextran degradation began to be seen at 50 °C [16]. In this study, LADNP showed a unique endothermic peak at 164.79 °C in 12.26 min. Previous studies claimed that a DSC analysis on dextran nanoparticles showed an endothermic peak at 165.77 °C in 12.46 min [6]. According to another report, praziquantel-dextran hydrogel displayed an endothermic peak at 138.7 °C [17]. The thermal stability of LADNP in oxygen was measured using TGA analysis. Figure 3B depicts the results of the TGA analysis of LADNP, showing a unique peak consistent with a degradation effect. Interestingly, from the thermogram, it can be understood that the initial weight loss in LADNP was observed at around 80 °C. At 210.78 °C, about 95% weight loss was observed, indicating that LADNP was thermo unstable at high temperatures. An earlier study suggested that the amikacin sulfate polymeric nanoparticles exhibit superior heat stability [18]. Below a temperature of 200 °C, a weight loss of less than 7% is seen. In addition, the slow weight loss continues up to 295 °C [18]. The results of the thermal analysis demonstrated that LADNP has a high thermostability.

### 3.3. XRD Analysis

**Figure 4:**
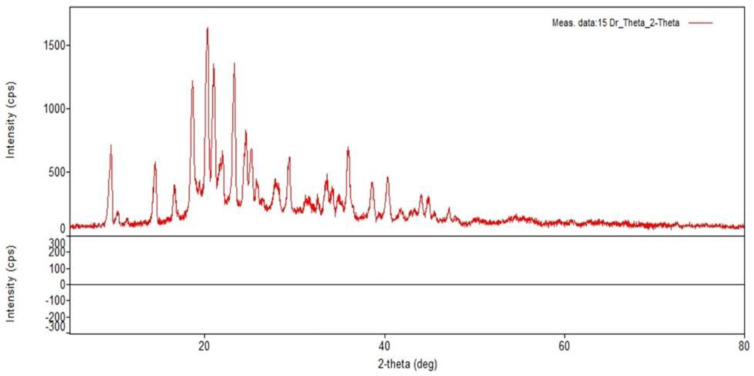
XRD analysis of lyophilized amikacin sulfate loaded dextran nanoparticles (LADNP). The diffractogram was obtained at 2*θ* in the range 2°–80°.

The discrete crystalline nanoparticle structure is characterized using XRD measurements. In the present study, XRD analysis at 2*θ* showed the presence of crystalline nanoparticles based on specific diffraction peaks at 9.6°, 10.4°, 11.4°, 18.9°, 20.3°, 24.4°, 28.2°, 33.2°, 38.9°, and 40.4°confirmed the unique design of the nanoparticles (Figure 4). An earlier study suggested that dextran nanoparticles showed specific diffraction peaks at 21.56°, 33.37°, 38.73°, 47.17°, 52.96°, and 58.42° [6]. According to the findings of a previous study, dextran-coated iron oxide nanoparticles exhibited a distinctive peak with maximum intensity at 35.79° [19]. In line with the earlier reported work, the present also showed a distinct, prominent peak at 20.3°, 24.4°, and 28.2° with maximum intensity. However, the unique peaks were extended up to 40.4°.

### 3.4. Loading and In Vitro Release Profile

**Figure 5:**
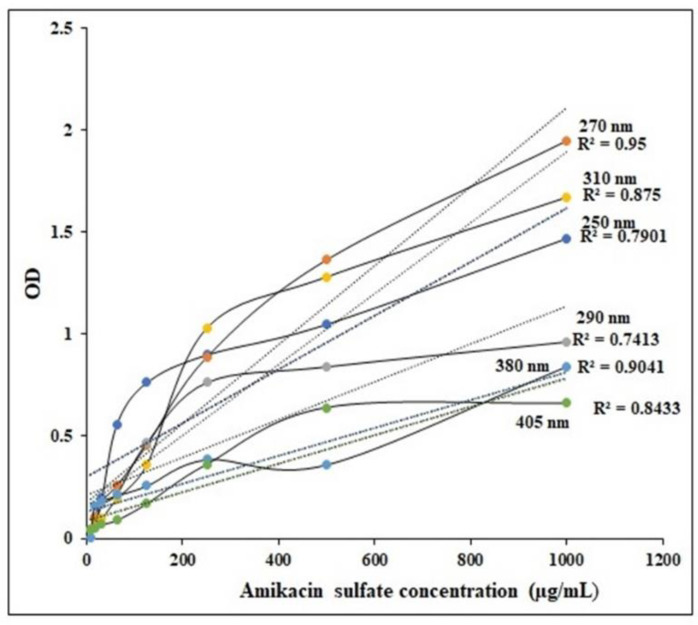
Standardization of Amikacin sulfate at various wavelengths.

This research presents a novel approach for standardizing amikacin sulfate in the UV region. The standard curve of amikacin sulfate at various wavelengths is depicted in Figure 5.

**Figure 6:**
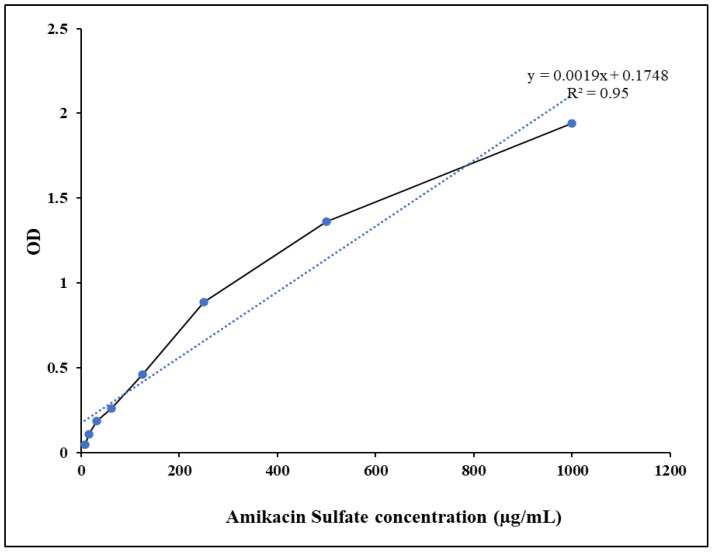
The standard curve of amikacin sulfate was determined at 270 nm.

According to the study, amikacin sulfate determination was most successful at 270 nm, where its linearity (R^2^) of 0.95 indicated compliance with Beers Lambert’s law (Figure 6).

**Figure 7:**
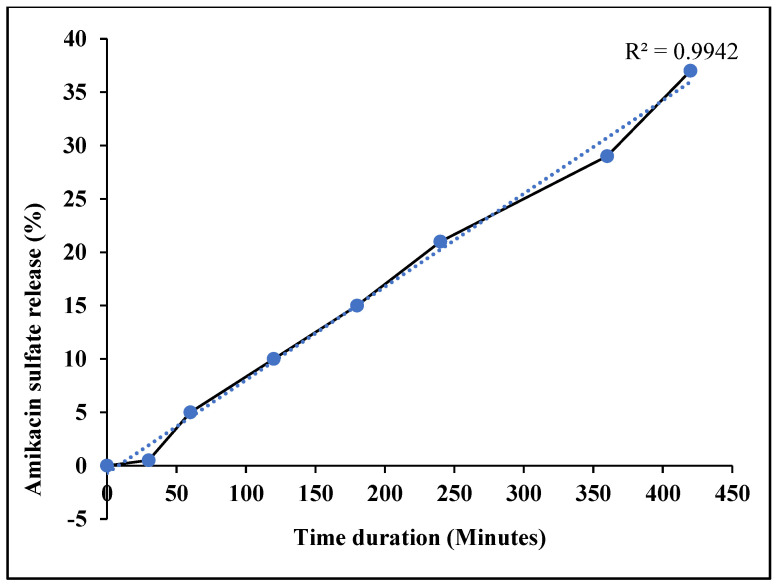
In vitro dissolution profile.

The current investigation demonstrated that amikacin sulfate was successfully loaded with 91.5 ± 1.2% loading. It is very clear that there was no initial burst release of amikacin sulfate, even though the cumulative percentage release of amikacin sulfate from LADNP into the medium was quite sustained. The amount of amikacin sulfate that was released was 0.5% in 30 min (Figure 7). However, in 60 min, there was a 5% release of amikacin sulfate. The drug release from LADNP between 60 and 120 min was 5%. However, the release between 120 and 180 min was 5%, while the release between 180 to 240 min was 6%. Interestingly, the drug release between 240 to 420 min was 16%. The release profile demonstrated strong linearity, as indicated by the R^2^ value of 0.9942. Amikacin sulfate was shown to be released from solid lipid nanoparticles at a rate of more than 80 percent in 10 days, according to an earlier report. Another study showed that 25% amikacin sulfate could be entrapped in thiolated chitosan nanoparticles for oral delivery, and drug release was determined in 18 h [20]. Recently, polylactide matrices loaded with amikacin sulfate were able to release the amikacin over 60 days [21]. On the other hand, according to these findings of their investigation, the current study released amikacin release much uniformly that, reached 37% release in 7 h, which will be more significant to optimize the dosage to once daily because the dosage frequencies vary with pathological effects.

### 3.5. Antibacterial Study

**Figure 8:**
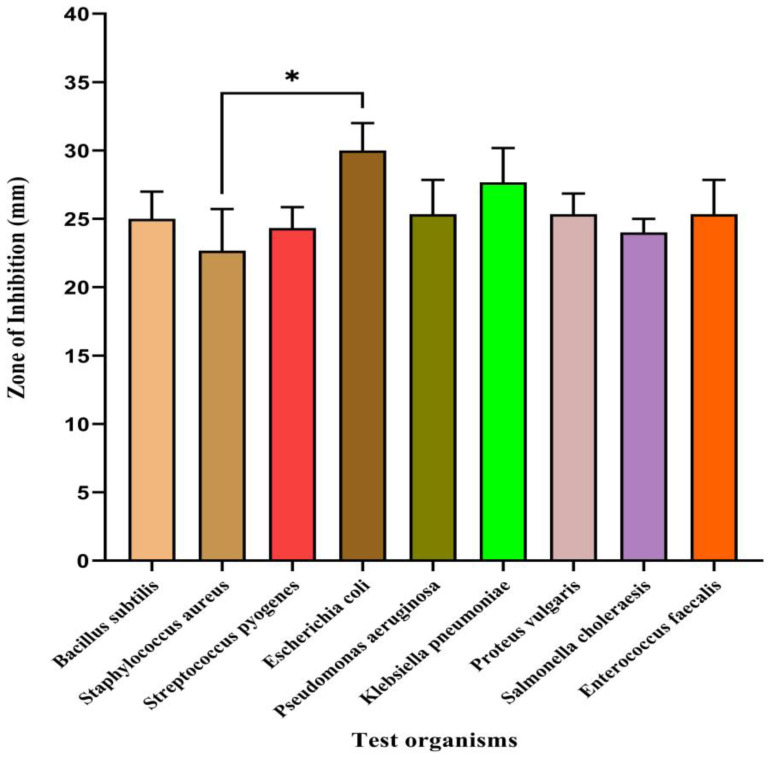
Antibacterial spectral study of lyophilized amikacin sulfate dextran nanoparticles against various human pathogenic bacteria. * Significant at *p* > 0.05.

**Figure 9:**
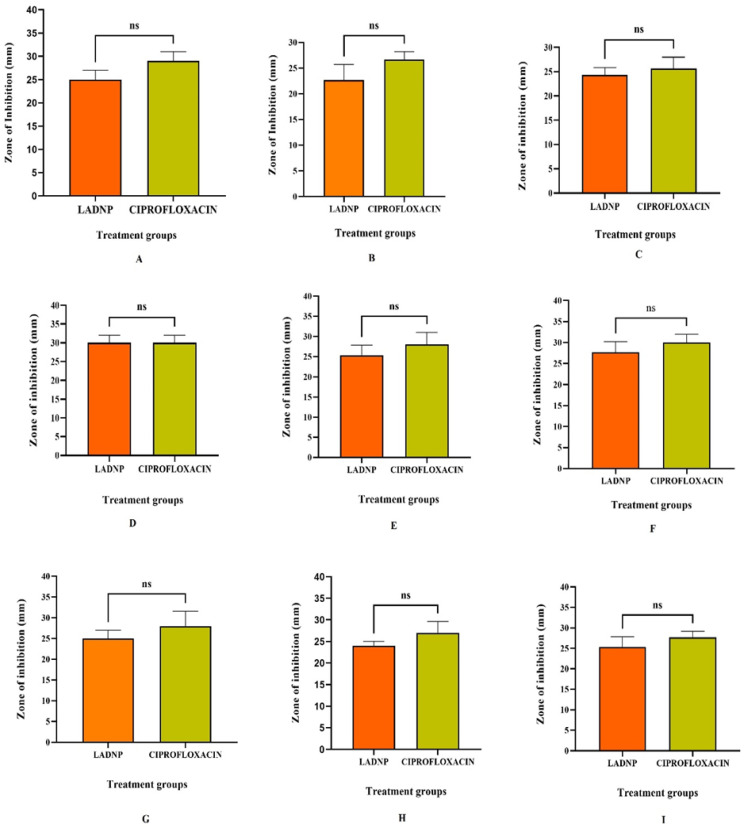
Comparative antibacterial spectral study. Amikacin sulfate dextran nanoparticles vs. Ciprofloxacin. (**A**) Against *Bacillus subtilis* at 2 × 10^5^ CFU/mL concentration (**B**) Against *Staphylococcus aureus* at 4 × 10^5^ CFU/mL concentration (**C**) Against *Streptococcus pyogenes* at 4 × 10^3^ CFU/mL concentration (**D**) Against *Escherichia coli* at 3 × 10^5^ CFU/mL concentration (**E**) Against *Pseudomonas aruginosa* at 2 × 10^3^ CFU/mL concentration (**F**) Against *Klebsiella pneumonia* at 2 × 10^4^ CFU/mL concentration (**G**) Against *Proteus vulgaris* at 3 × 10^3^ CFU/mL concentration (**H**) Against *Salmonella choleraesuis at* 2 × 10^4^ CFU/mL concentration (**I**) Against *Enterococcus facalis* at 4 × 10^3^ CFU/mL concentration. ns: non-significant.

The LADNP demonstrated promising broad-spectrum efficacy against Gram-positive and Gram-negative bacteria (Table 2). The study explored the potential antibacterial effects of LADNP on the screened organisms. Depending on the organism, the MICs of LADNP against the tested bacterial species ranged from 102.6 ± 5.2 to 260 ± 1.63 g/mL. Consequently, a concentration of 260 g/mL LADNP was used in this study’s antibacterial spectrum studies. The surface charge and size of nanoparticles are crucial parameters for targeting bacterial cells because they impact the extent of nanoparticle penetration into bacterial cells. In the present study, the broad-spectrum efficacy of LADNP was observed against various bacterial organisms. The study showed that LADNP exhibited maximum activity against *Escherichia coli*, which is significant at *p* < 0.05 level when compared to *Staphylococcus aureus*. However, the activity against the rest of the bacterial organisms is equally effective (Figure 8). In a previous study, it was revealed that dextran sulfate nanoparticles were more effective against *Escherichia coli* and had robust broad-spectrum action against Gram-positive and Gram-negative bacteria [6]. A study published in 2016 demonstrated that dextran sulfate silver nanoparticles produced potent antimicrobial activity against a wide range of microorganisms, including *Bacillus cereus*, *Bacillus luteus*, *Bacillus subtilis*, *Staphylococcus aureus*, *Escherichia coli*, *Pseudomonas aeruginosa*, *Listeria monocytogenes*, *Klebsiella pneumoniae*, and *Proteus vulgaris* [22]. Dextran, which has undergone chemical modification, has been found to have powerful antibacterial properties, particularly against *Pseudomonas aeruginosa* and *Staphylococcus aureus* [23]. According to a recent study, amikacin-loaded niosome nanoparticles enhance amikacin efficacy against antibiotic-resistant *Klebsiella pneumonia* strains by inhibiting the development of biofilms. The recent development of a liposomal formulation of amikacin revealed that the formulation was effective against *Mycobacterium avium* and *Mycobacterium abscessus* in infected macrophages [24]. An early study indicated that a nanoscale liposomal formulation of amikacin for nebulization and inhaled administration was penetrated to sputum and biofilm, indicating a possible benefit against *Pseudomonas* infection in the lungs of cystic fibrosis patients [25]. In the present study, LADNP exhibited a broad spectrum of activity against Gram-positive and Gram-negative bacteria (Table 2). When compared to the efficacy of ciprofloxacin, LADNP’s antibacterial activity spectrum was almost identical and non-significant at *p* < 0.005 (Figure 9). The importance of nano formulations as strong antibacterial agents against screening bacterial pathogens such *Staphylococcus aureus*, *Escherichia coli*, and *Pseudomonas aeruginosa* was suggested in earlier investigations. As a result, nanoparticles offer some benefits when it comes to treating bacterial infections [26,27]. The enhanced permeability and retention effect, often known as the EPR effect, is critical in targeting bacterial cells. This effect is determined by the size of the nanoparticles as well as their ZP [28]. It is possible to gain insight into the ease with which LADNP can diffuse across bacterial membranes by considering both the ZP and the particle size of the LADNP. Therefore, the diffusion of LADNP into bacteria is possible if the size of the nanoparticles and the surface charges are optimized. It has been found that nanoparticles with diameters ranging from 50 to 200 nm easily pass through the membrane of bacteria. It’s interesting to note that LADNP’s size ranged from 260 to 320 nm, indicating a reasonable particle size for bacterial cell targeting. However, despite the fact that the average zeta size was identified as 317.9 z. d.nm, the LADNP displayed outstanding antibacterial action.

## 4. Conclusions

This study used the ionic gelation technique to prepare amikacin-loaded dextran nanoparticles. The formulation proved successful in physicochemical qualities, achieved amikacin sulfate loading, sustained release from LADNP, and triggered a broad spectrum of antibacterial activities. These results indicate that injectable LADNP is a promising antibacterial agent, an innovative therapeutic formulation for treating bacterial infections. Based on the outcome of current research, dextran sulfate nanoparticles have beneficial to be utilized for a variety of antibiotics to combat infectious diseases. However, it is necessary to conduct additional research on the formulation and evaluation process to overcome the lacuna we experienced during the present study. Therefore, the future perspective of the present study is to optimize formulation concerning the strength of LADNP per injectable dosage form. Furthermore, accelerated stability studies are essential after optimizing product development. Moreover, the LADNP must be screened for both potencies and toxicity studies by challenging methods through in vivo analysis.

## Figures and Tables

**Table 1:** Dynamic light scattering analysis of Amikacin sulfate loaded dextran sulfate sodium nanoparticles.

Zeta Potential (mV)	Zeta Average Size (z.d.nm)	Size (d.nm)	% Intensity	Y Intercept	PDI	% PDI	Pd (d.nm)	% Mass (d,nm)	Conductivity (mS/cm)
−20.9 ± 8.35	317.9	259.3 ± 73.52	90.2	0.854	0.256	67.7	215.2	57.63	2.36

**Table 2:** Antibacterial study.

Organisms	Concentration CFU^#^/mL	MIC (µg/mL)	Zone of Inhibition (mm)	
			LADNP	Ciprofloxacin (50 µg/mL)
*Bacillus subtilis*	2 × 10^5^	151.3 ± 2.5	25 ± 2	29 ± 2
*Staphylococcus aureus*	4 × 10^5^	201 ± 2.4	22.67 ± 3	26.67 ± 1.5
*Streptococcus pyogenes*	4 × 10^3^	260 ± 1.63	24.33 ± 1.5	25.67 ± 2.3
*Escherichia coli*	3 × 10^5^	102.6 ± 5.2	30 ± 2	33.67 ± 1.5
*Pseudomonas aruginosa*	2 × 10^3^	121 ± 2.9	25.3 ± 2.5	28 ± 3
*Klebsiella pneumonia*	2 × 10^4^	123.3 ± 1.2	27.67 ± 2.5	30 ± 2
*Proteus vulgaris*	3 × 10^3^	148 ± 1.6	25.33 ± 1.5	28 ± 3
*Salmonella cholerasis*	2 × 10^4^	151.3 ± 3.4	24 ± 1	27 ± 2.7
*Enterococcus facalis*	4 × 10^3^	122.3 ± 2.1	25.3 ± 2.5	27.67 ± 1.5

Each value is the mean of 3 batches with a standard deviation. The statistical analyses were performed using the Prism 9, Graph Pad Instat software system, USA. ^#^ CFU—Colony Forming Unit; MIC: Minimum Inhibitory Concentration; LADNP: Lyophilized Amikacin sulfate-loaded Dextran Nanoparticles.

## Data Availability

Data is contained within the article.
